# The relationship of central corneal thickness with the status of diabetic retinopathy

**DOI:** 10.1186/s12886-020-01411-2

**Published:** 2020-06-08

**Authors:** Handan Canan, Nedime Sahinoglu-Keskek, Rana Altan-Yaycioglu

**Affiliations:** 1 Department of Ophthalmology, Baskent University, School of Medicine, Dadaloğlu Mah, Serinevler, 01250 Adana, Turkey; 2 Department of Ophthalmology, Acibadem Adana Hospital, Adana, Turkey

**Keywords:** Central corneal thickness, Diabetic retinopathy

## Abstract

**Background:**

To compare central corneal thickness (CCT) values measured by three different devices: slit-scanning topography (SST), ultrasonic pachymetry (UP), and optical coherence tomography (OCT) in diabetic eyes and compare the CCT values in patients with and without diabetic retinopathy.

**Methods:**

Ninety-six patients with diabetes mellitus were included in this prospective study and divided into two groups according to the presence of diabetic retinopathy, as Group I with retinopathy and Group II without. The CCT of 96 eyes was measured by three different devices; SST (Orbscan II), UP and OCT. The results of CCT measurements with three different devices were compared. Also, the intergroup differences in CCT measurements were evaluated.

**Results:**

The CCT was statistically insignificantly different between the two groups. Although the three methods of CCT measurements correlated well with each other, SST showed significantly (*p* < 0,0001) higher CCT results compared to both UP and OCT.

**Conclusions:**

According to our results, neither the duration of DM nor the presence of diabetic retinopathy did have a significant effect on the CCT. The CCT values obtained with three devices were all in correlation. However, the results of SST were significantly higher compared to the other two. Our findings emphasize the value anterior segment OCT in CCT measurements, since it is a non-contact method and correlate very well with UP.

## Background

Diabetes mellitus (DM) is the leading cause of blindness worldwide as a result of complications related to retinopathy. The disease is also associated with a variety of corneal disorders such as punctuate epithelial keratopathy, recurrent corneal erosions, persistent epithelial defects, and endothelial damage [1, 2]. The metabolic status of the cornea is affected by the changes in blood glucose levels [3–6]. Chronic metabolic stress caused by hyperglycemia has shown to lead in alterations at cellular level affecting the corneal endothelial cells, which are responsible in maintaining stromal hydration by actively removing water, namely endothelial pumping mechanism. Thus, it is possible that central corneal thickness (CCT) may change in accordance with the irregularities of blood glucose levels [7].

In present study, our aim was to analyze CCT in diabetic patients with or without retinopathy, with the hypothesis that patients with diabetic retinopathy might also show increase in CCT related to metabolic changes of the cornea. We also aimed to investigate the effects of other factors on CCT, such as hemoglobin A1c (HbA1c) levels, the duration of DM, and/or the stages of diabetic retinopathy. Besides, the results of CCT measured with three different devices were compared.

## Methods

In this prospectively designed controlled clinical trial, 96 consecutive Caucasian patients, with the diagnosis of DM, were included. One eye of each subject was randomly selected for analysis. This clinical study was conducted according to the principles of Declaration of Helsinki. Institutional Review Board Approval was obtained (KA 15/46). All patients were informed on the risks and benefits of the procedure and written informed consent was obtained from all participants. The exclusion criteria were pregnancy, history of trauma, previous refractive surgery, corneal abnormalities (such as corneal edema, opacity, dystrophy), refractive errors greater than three diopters, contact lens use, glaucoma, history of photocoagulation in the last 3-months, and history of long-term topical ophthalmic medication use.

Main outcome measure of the study was to evaluate the relationship of retinopathy and HbA1c with CCT. The second outcome measure was to compare the results of three corneal pachymetry devices.

All patients underwent detailed ophthalmologic examination. Two clinicians (HC and NSK) examined all patients, and they were masked to the diagnosis of each other. Diabetic retinopathy was classified according to the criteria of Early Treatment Diabetic Retinopathy Study (ETDRS) [8]. Following the dilated fundus evaluation, the diagnosis was concluded by the agreement on the diagnosis. Subjects were assigned into 2 groups according to the presence of diabetic retinopathy: Patients, who were diagnosed with diabetic retinopathy were included in the first group (Group I, study group), and patients with DM but no sign of diabetic retinopathy were included in the second group (Group II, control group). Age, gender, visual acuity, intraocular pressure (IOP), duration of diabetes, data on previous laser treatment, and HbA1c levels were recorded.

In addition to routine examination, central corneal thickness mesurements were performed between 11 am and 2 pm with three different devices. First, slit-scan topography (SST, Orbscan Topography System, Orbscan II, Bausch & Lomb, France) measurements were obtained. Second, anterior segment optical coherence tomography (OCT, OptovueRTVue 100–2, Fremont, CA) images were taken. And third, ultrasonographic pachymetry (UP, UP-1000 Ultrasonic Pachymetry, Nidek Co, Aichi, Japan) was performed following topical anesthesia. Five consecutive measurements were obtained with UP, and the average of these five readings was recorded as the CCT value.

### Statistical analysis

Statistical analysis was performed using the statistical package SPSS v 23.0. For each continuous variable; normality was checked by Kolmogorov test. All numerical data were expressed as median values (minimum-maximum) or as proportions. Comparisons between the groups were performed using Student t-test in case of normally distributed data. The categorical variables of the groups were analyzed by using the Chi square test. Correlations were tested by Inter-rater correlation test. Spearman’s correlation coefficients were interpreted as either excellent relationship r ≥ 0.91; good 0.90 ≤ r ≥ 0.71; fair 0.70 ≤ r ≥ 0.51; weak 0.50 ≤ r ≥ 0.31; little or none r ≤ 0.3 [9]. The agreement of three devices were shown on Bland-Altman plots. A *p* value of 0.05 was taken as the level of significance.

Sample calculation was based on the publication of “Corneal Thickness Measurements With Contact and Noncontact Specular Microscopic and Ultrasonic Pachymetry” (American Journal Of Ophthalmology, October 2011) [10]. According to this study, at least 40 patients should be included in the study. So we included more than 40.

## Results

There were 47 patients in Group I (27 female, 20 male) and 49 patients in Group II (27 female, 22 male). The demographic features of patients are shown on Table 1. The mean age ± standard deviation (SD) was statistically significantly higher in Group I compared to Group II (*p* = 0.003). Considering the refractive errors, median spherical equivalent (SE) was 0.00 in Group I and 0.50 in Group II, and the refractive errors were similar in the two groups (*p* = 0.682). The intraocular pressure (IOP) values were insignificantly different between groups (*p* = 0.241). The HbA1c levels were slightly higher in Group I, however the difference was statistically insignificant (*p* = 0.121). The duration of diabetes was 16.89 ± 4.94 years in Group I, and 8.73 ± 5.71 years in Group II, which was statistically shorter in Group II (*p* = 0.0001). In Group I, 30 patients had non-proliferative diabetic retinopathy (NPDR) and 17 patients had proliferative retinopathy (PDR). Twenty-two patients had history of argon laser photocoagulation; the remaining 25 had no history of photocoagulation.

**Table 1:** The descriptive features of patients according to the groups are shown as mean ± SD (range) values

	Group I (*n*=47) (range)	Group II (*n*=49) (range)	p
**Female/Male (n)**	20 / 27	22 / 27	0.840
**Age**	58.45±10.51 (37-82)	52.61±8.15 (37-70)	0.003*
**SE**	0.03 ± 0.91 (-2.00 D - +2.00 D)	0.11 ± 1.00 (-2,50 D - +2,00 D)	0.682
**IOP (mmHg)**	17.62 ±3.57 (11-26)	16.82±3.06 (8-24)	0.241
**DM duration (years)**	16.89±4.94 (10-30)	8.73±5.71 (2-24)	0.000*
**HbA1c**	8.44 ± 1.69 (5.88-13.27)	7.84 ± 2.03 (5.08-13.36)	0.121

Group I included patients with diabetic retinopathy, Group II included patients without diabetic retinopathy. Probability value (p) was accepted as significant if less than 5%, and was assigned with “*”

*SE* spherical equivalent, *IOP* intraocular pressure, *DM* diabetes mellitus, *HbA1c* glycated hemoglobin A1c, percent of total hemoglobin

**Fig. 1:**
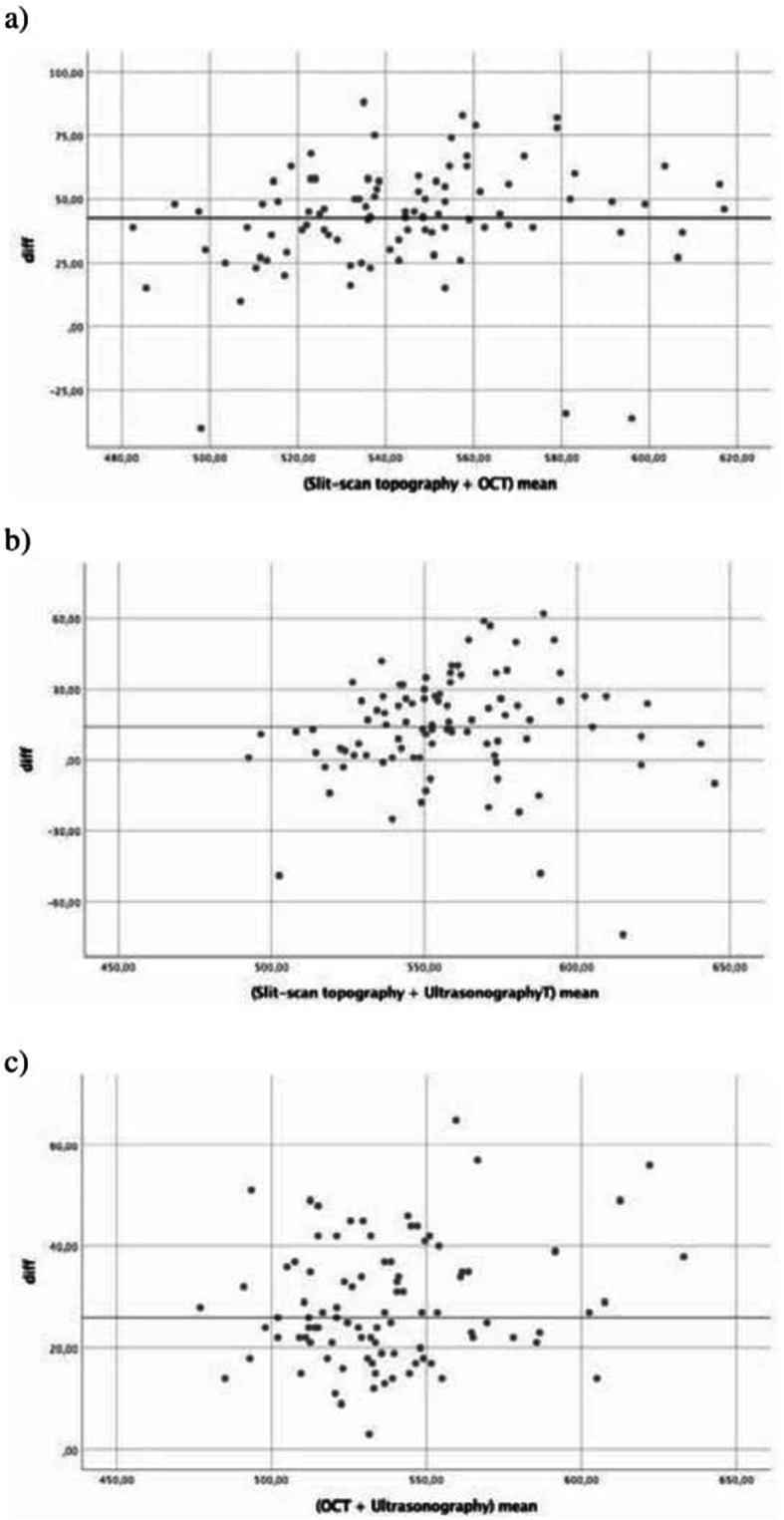
Bland-Altman graphs showing the results of pachymetry devices in present study. **a** Slit-scan topography (SST) vs optical coherence tomography (OCT); **b** SST vs. ultrasonography (UP); **c** OCT vs. UP

The CCT values measured with each device are shown on Table 2. The values showed no difference related to the presence of retinopathy, and between the two groups no statistically significant difference was found with any device (*p* > 0.05). The mean SST pachymetric values were higher compared to OCT and UP values. Although, the measurements were in correlation with three pachymetric devices, the correlation of OCT and UP was stronger (*r* = 0.92, *p* = 0.0001) compared to SST with the other two instruments (*r* = 0.76, *p* = 0.0001, for both UP and OCT) (Table 3). The agreement of the measurements is shown on Bland Altman plots (Fig. 1 a,b,c). The mean CCT values of each subgroup of patients, with non-proliferative and proliferative diabetic retinopathy, are shown on Table 2. There was also no significant difference in CCT between these patients (*p* = 0.239). According to the analysis, CCT was not correlated with HBA1c (*p* = 0.121), IOP (*p* = 0.241), duration of diabetes (*p* = 0.440), retinopathy severity (*p* = 0.239), and history of previous laser therapy (*p* = 0.406).

**Table 2:** The mean central corneal thickness in Group I, Group II, and subgroups in Group I

	Group I	Group II	p	Subgroups in Group I		p
				NPDR	PDR	
**Number of patients**	47	49		30	17	
**CCT–OCT**	524.13±31.21	521.71±27.58	0,689	528.20 ± 29.16	516.94± 34.25	0.239
**CCT–SST**	562.83 ±32.69	568.10±32.51	0,430	567.57±35.49	554.47 ±25.95	0.190
**CCT–UP**	551.77 ±33.25	551.10±29.64	0,918	556.07±31.18	544.18 ±36.33	0.243

Group I included patients with Diabetic retinopathy, Group II included patients without diabetic retinopathy. Subgroups were divided according to the proliferation as NPDR and PDR. Probability value (p) was accepted as significant if less than 5%, and was assigned with “*”

*NPDR* nonproliferative diabetic retinopathy, *PDR* proliferative diabetic retinopathy, *CCT* central corneal thickness, *OCT* optical coherence tomography, *SST* slit scan topography, *UP* ultrasonography

**Table 3:** The correlations of three different methods of central corneal thickness measurements are shown. First, values of all patients were investigated for correlation. Later, separate calculations for Group I, and II were performed

**All patients**		**SST**	**UP**
**OCT**	r	0,76	0,92
	p	0,0001*	0,0001*
**SST**	r		0,76
	p		0,0001
		**SST (% 95 CI)**	**UP (% 95 CI)**
**Group I**			
**OCT**	r	0,67 (0,48-0,80)	0,93 (0,87-0,96)
	p	0,0001*	0,0001*
**SST**	r		0,66 (0,47-0,80)
	p		0,0001*
**Group II**			
**OCT**	r	0,88 (0,78-0,92)	0,92 (0,87-0,96)
	p	0,0001*	0,0001*
**SST**	r		0,87 (0,68-0,92)
	p		0,0001*

Group I included patients with Diabetic retinopathy, Group II included patients without diabetic retinopathy. Probability value (p) was accepted as significant if less than 5%, and was assigned with “*”

*OCT* optical coherence tomography, *SST* slit scan topography, *UP* ultrasonography, *r* rho, correlation coefficient, *p* probability value, *CI* confidence interval

## Discussion

Corneal thickness can be associated with several factors like age, race, sex, and systemic diseases such as DM [2, 4, 11, 12]. Diabetes mellitus is characterised by chronic metabolic stress caused by hyperglycemia that might lead to alterations in corneal epithelium, stroma and endothelium [13]. In diabetic patients,corneal endothelial dysfunction and increased corneal hydration may affect corneal thickness [2, 4–7, 14], and this might be related to endothelial problems, possibly resulting in endothelial decompensation following cataract surgeries. In present study, we aimed to evaluate the corneal thickness in patients with and without diabetic retinopathy, and found no correlation between the two (*p* = 0,689). We neither have found correlation of CCT with disease duration, severity of retinopathy,and previous laser therapy. Besides, we compared three devices, namely SST, OCT and UP methods, and found that OCT and UP were in better correlation. Although in correlation with the other two, SST showed higher values. In present study, there was a significant age difference between the two groups, patients in Group I were older (*p* = 0.003). The duration of diabetes was also longer in Group I (*p* = 0.0001). Probably, the time interval following diagnosis of diabetes, thus duration of diabetes was longer in patients with diabetic retinopathy eventually leading elderly age in this group. Several studies reported decreasing values of CCT in relation to older age [12, 15]. On the other hand, in a study including quite large population (5158 patients, age range 17 to 83 years) Prasad et al. showed that CCT was not affected by age [16]. In accordance with that, we also found no correlation between age and CCT.

An accurate measurement of corneal thickness is important in many ophthalmic circumstances: accurate IOP measurements, preoperative evaluation in ocular surgeries, assessment of postoperative healing process, planning of keratorefractive procedures, and evaluation of corneal endothelial function in diabetic patients. Currently, several different methods are being used to measure the thickness of the cornea. Herein, we planned to compare CCT values measured with three relatively most commonly used devices, in order to determine which device will be more reliable in diabetic eyes. Although all three methods correlated well and were in aggreement, SST measurements were higher compared to the other two (*p* = 0.0001). Previously, in normal corneas a correlation between UP and SST (Orbscan II) was shown [17, 18]. As being a non-contact method, Orbscan was reported as a repeatable technique; however, the device systematically overestimated CCT compared with other devices [19–21]. With Orbscan device applying an acoustic equivalent correction factor provided measurements of CCT similar to US in normal subjects. In Gonzalez-Perez et al’s study, with no correction factor applied the Orbscan significantly overestimated CCT by 32 ± 15 μm when compared with US [21]. There are several other studies suggesting the use of acoustic equivalent correction factor, since without it the results were approximately 23–30 μm higher than the UP [19–21]. In present study, we have used SST without the acoustic correction factor, which we believe might explain the overestimated results we measured with this device. Optical coherence tomography (OCT) was first introduced for the analysis of posterior segment. In relatively recent years, anterior segment imaging also became available with this device. Later on, it is being used for cross-sectional corneal analysis, corneal thickness measurements, and also quantitative analysis of the cornea [22, 23]. CCT measurements made with the OCT system correlate very well with the results of other conventional methods. Additionally, it does have the advantage of being a non-contact method; thus it can be used soon after corneal surgery [22, 23]. Our results correlated well with previous studies, showing excellent correlation of CCT measurements with OCT and UP (*r* = 0.96).

Glycemic status (HbA1c) should be considered when examining the eye of diabetic patients. HbA1c reflects changes in glucose concentrations over a two to 3 month period and reflects the patient’s general tendency to diabetes control [7, 14]. Since poor glycemic control (high HbA1c levels) is associated with an increased risk of diabetic complications, the American Diabetes Association recommends that the mean HbA1c value should be kept below 7% to prevent diabetic micro and macrovascular complications [24]. In present study, we aimed to investigate the potential effects of diabetic retinopathy status and serum HbA1c levels on CCT, and found no correlation between the two parameters. Ozdamar et al. also did not found statistically significant correlations between variables HbA1c and CCT in the diabetic subgroups [14].

Several studies investigated the relationship between corneal thickness and diabetes and reported variable results (Table 4). Some authors reported increased CCT in association with hyperglycemia [2, 4, 6]. Lee et al. found that CCT was significantly higher in diabetic patients (588.2 ± 2.7 μm) compared to the control group (567.8 ± 3.8 μm) [2]. Ozdamar et al.also found higher CCT values in patients with DM compared with control group, and also showed that patients with PDR had thicker CCT than those with NPDR and without retinopathy. However, the difference was statistically insignificant [14]. On the other hand, some other studies reported no significant difference in corneal thickness in patients with diabetes [3, 25, 26]. Inoue et al. [26] and Wiemer et al. [3] found no significant difference in CCT between diabetics and controls. Keoleian et al. also reported no difference in CCT between subjects with DM and healthy controls [25]. In present study, we evaluated whether the presence of retinopathy affected CCT, and according to our results no statistically significant difference was found between patients with (Group I) and without (Group II) diabetic retinopathy. There was also no statistically significant difference between PDR and PDR subgroups. In contrast to our results, Busted et al. found an association between the level of retinopathy and CCT, and reported that increased corneal thickness may be an indicator of the risk of retinal complications in diabetic individuals [5]. Lee et al. also found that CCT was significantly higher in patients with longer duration of DM, over 10 years, (595.9 ± 4.2 μm) compared to the ones with shorter, less than10 years of duration (582.2 ± 3.7 μm) [2]. In present study, we did not found any correlation with disease duration. Similar to our study, Wiemer et al. also reported no association between disease duration, level of retinopathy and CCT [3]. Busted et al. also reported no significant correlations between diabetes duration, blood glucose levels, or use of insulin and CCT [5]. The differences in disease duration and CCT values are probably the result of patients’ metabolic status included in the studies.

**Table 4:** Characteristics of the studies evaluating the CCT in diabetic patients

References (Author, year)	Eyes (N)		CCT Mean ± SD		Method of CCT measurement	Significant correlation
	DM group	Control group	DM group	Control group		
Lee at al. 2006 [2]	200	100	588.2±72.7 μm	567.8±73.8 μm	Ultrasound pachymetry	Yes
Wiemer et al. 2007 [3]	102 (DM type 1)	69	0.586 ±0.003 mm	0.578± 0.004 mm	Topcon SL-45 Scheimpflug camera	No
	101( DM type 2)		0.578 ±0.003 mm			
Busted et al. 1981 [5]	Diabetics without PR	67	0.544±0.028 mm	0.527±0.028 mm	Modified Haag-Streit pachometer	Yes
	Diabetics with PR		0.566±0.027 mm			
	(total 81)					
Keoleian et al. 1992 [25]	14	14	0.56 ± 0.02 mm	0.56 ± 0.04 mm	Pachymetry	No
Özdamar et al. 2010 [14]	100	145	564±30 μm	538±35 μm	Biopachymeter (Tomey, Nagoya)	Yes
Inoue et al. 2002 [26]	99	97	538± 36 μm	537 ± 38 μm	Ultrasonic pachymeter (AL-2000; Tomey, Nagoya)	No

*CCT* central corneal thickness, *DM* diabetes mellitus, *N* number, *SD* standard deviation, *PR* proliferative retinopathy

Our study had several limitations. First, lack of blood glucose level evaluation while the measurements were taken might have had an addition to our results. And second, a control group of healthy subjects is lacking. However, since we aimed to investigate the relationship of retinopathy with CCT, we believe that control group of healthy subjects was not necessary.

## Conclusion

Inconclusion, we did not find an increased CCT in DM regardless of the severity of the retinal disease. Although the results were in correlation with the other two methods, the CCT measurements with SST were overestimated compared to the other two. Our findings emphasize the value anterior segment OCT in CCT measurements, considering that it is a non-contact method and it does correlate very well with UP. Further studies in larger patient groups are necessary to support our results, and to investigate whether corneal thickness could be an indicator of the metabolic status of DM.

## Data Availability

The datasets used and/or analyzed during the current study are available from the corresponding author on reasonable request.

## Abbreviations

SST: Slit scanning topography
OCT: Optical coherence tomography
CCT: Central corneal thickness
DM: Diabetes mellitus
IOP: Intraocular pressure
UP: Ultrasonic Pachymetry
ETDRS: Early Treatment Diabetic Retinopathy Study
HbA1c: Glycated hemoglobin A1c, percent of total hemoglobin
PDR: Proliferative Diabetic Reitinopathy
